# The effect of Mind Body Medicine course on medical student empathy: a pilot study

**DOI:** 10.3402/meo.v21.31196

**Published:** 2016-06-27

**Authors:** Allen K. Chen, Anagha Kumar, Aviad Haramati

**Affiliations:** 1 Georgetown University School of Medicine, Washington, DC, USA; 2 Medstar Health Research Institute, Georgetown University Medical Center, Washington, DC, USA; 3 Department of Biochemistry, Molecular and Cellular Biology, Georgetown University Medical Center, Washington, DC, USA; 4 Department of Medicine, Georgetown University Medical Center, Washington, DC, USA

**Keywords:** empathy, stress, medical education, mind body medicine

## Abstract

**Introduction:**

Empathy among medical practitioners has been shown to affect patient care and outcomes. Factors such as stress and depression are known to have a negative impact on medical student empathy. Approaches such as mindfulness, meditation, and other mind–body techniques can enhance empathy and reverse burnout symptoms. In the present study, we evaluated impact of Mind Body Medicine (MBM) course on perceived stress and empathy on first-year medical students.

**Methods:**

Thirteen first-year medical students in total self-selected into MBM (experimental) and seven non-MBM (control) groups completed a prospective, pre- and post-test analysis, using the Jefferson Scale of Physician Empathy – Students (JSPE-S), Perceived Stress Scale (PSS), and Personal Health Questionnaire (PHQ) to evaluate empathy, stress, and depression, respectively.

**Results:**

Our results showed an increase in stress, as well as a decrease in empathy, in both MBM and non-MBM groups throughout the course of the study.

**Conclusion:**

Our study demonstrated that the inverse relationship increased stress and decreased empathy among first-year medical students and participation in the MBM course did not attenuate the changes. However, a statistically significant rise in the depression score in the non-MBM group was not observed in the MBM group.

A well-developed patient–physician relationship has been shown to improve patient health outcomes and compliance (1, 2). Being empathic is one of the many factors contributing to enhancing the patient–physician relationship (3). In the clinical setting, empathy is defined as the ability to feel the patient experience to form better understanding (4). Empathy is beneficial to the provider as well, as it corresponds to better job satisfaction as well as improved clinical practice (1, 2, 4). Studies have shown that feelings of stress and depression have an inverse relationship with empathy among health care professionals and lead to lower quality of care and an increase in medical errors (5–7).

It is well known that medical students have high rates of stress and depression (8–14) and show decreases in empathy during training (1, 5, 15–18). Furthermore, a statistically significant percentage of medical students exhibit burnout and show increased signs of depression during medical school (1, 19). To address these challenges, many medical schools are developing curricular interventions to better prepare medical students for the rigors of the profession.

With these goals in mind, the Mind Body Medicine (MBM) program at Georgetown University School of Medicine offers a 11-week elective course to first-year medical students involving experiential introduction to practices such as meditation, guided imagery, and journal writing within small group interactions (19). The primary objective is to introduce students to MBM skills that foster self-awareness and help them better cope with stress. Each group of 10 students is facilitated by two trained faculty members. The program was started by a grant provided by Georgetown University School of Medicine in 2001 and a larger award from the National Institutes of Health (11).

Previous studies of the MBM course at Georgetown University have reported that participants had reduced perceived levels of stress and physiologic stress biomarkers and increased levels of empathy (9, 11–14). In the present study, we used different instruments to assess whether stress and depression in medical students would be affected by participation in the MBM course.

## Methods

This was a prospective, pre- and post-test quasi-experimental study. The study population consisted of medical students in the class of 2018 who received the Jefferson Scale of Physician Empathy – Students (JSPE-S), the Perceived Stress Scale (PSS), and Personal Health Questionnaire (PHQ). Our inclusion criteria were first-year medical students enrolled in the class of 2018.

We opted to use the JSPE-S (2, 15, 19) which is a validated instrument widely used in medical students. The JSPE was specifically designed for medical students (the S-version) in context of medical education and patient care (15). The PSS is a 14-item questionnaire answered on a five-point frequency scale. The PSS serves to measure each individual's perceived frequency of stress and has been widely used (11–13). The verified measure for depression is the PHQ. The PHQ is a nine-item questionnaire that focuses on symptoms of depression that the subject may experience. The total score is out of 27 points, and a clinical diagnosis of depression may be made if the subject answers 2 or 3 for 5 or more symptoms (20, 21). Copies of the JSPE were obtained with permission from the Center for Research in Medical Education and Health Care at Jefferson University. The PHQ and PSS are available in the public domain.

Survey participants were given unique identifiers. Demographics collected were age, gender, and race/ethnicity. The above demographics were collected prior to starting the MBM course. First-year medical students not participating in MBM course served as controls, while first-year medical students participating in MBM course served as the experimental group. After obtaining IRB approval, the JSPE-S, PHQ, and PSS were administered to first-year medical students at the beginning of their fall semester of the first year. This allowed us to measure baseline levels of stress, depression, and empathy and to evaluate any confounding variables. The students self-selected for the MBM course program, which consisted of approximately 60–80 students out of a class of approximately 180 students. Toward the end of the first year, the instruments were administered once again.

## Results

At the beginning of their fall semester (August 2014), 122 first-year medical students completed the surveys; however, only 27 completed the post-test surveys upon conclusion of their first year (May 2015). The data were matched with identifiers and results that did not include both a pre- and post-test were not included.

Ultimately, there were 20 individuals in this dataset. Of those, 7 were not enrolled in the MBM course and 13 were. We conducted Fisher's exact test to test whether enrollment in the MBM course was independent of the demographic characteristics. We concluded that enrollment in the MBM course was in fact independent of gender, ethnicity, race, marital status, and religion, respectively (*p*-values >0.05).

Using the Kolmogorov–Smirnoff test, a non-normal distribution (*p*-values<0.05) was observed. Therefore, a Wilcoxon Signed Rank test was used to test for pre–post differences within the MBM and non-MBM groups. The Wilcoxon Rank Sum test was used to make between-group comparisons at baseline and after completion of the course.

**Fig. 1:**
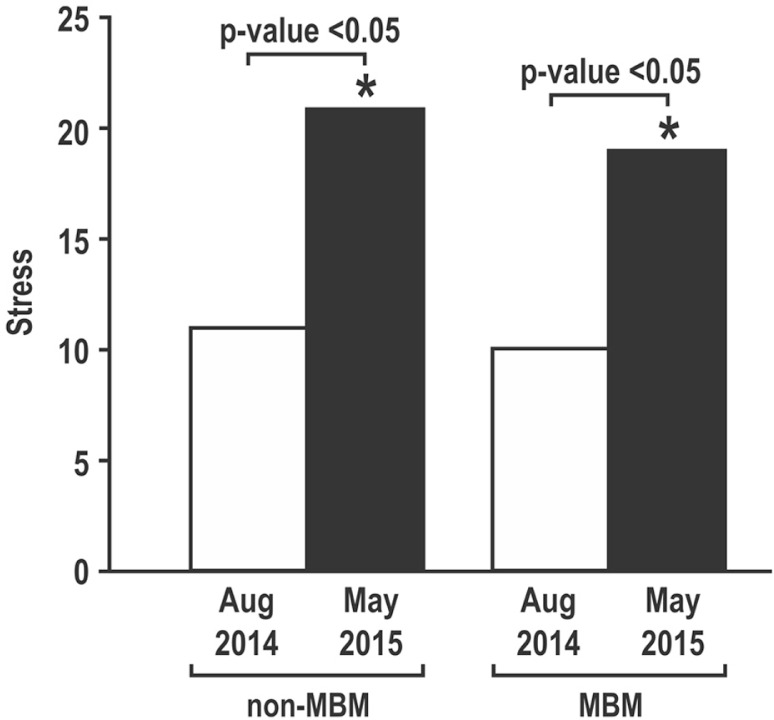
Scores on the Perceived Stress Scale (PSS) in first-year medical students.

**Fig. 2:**
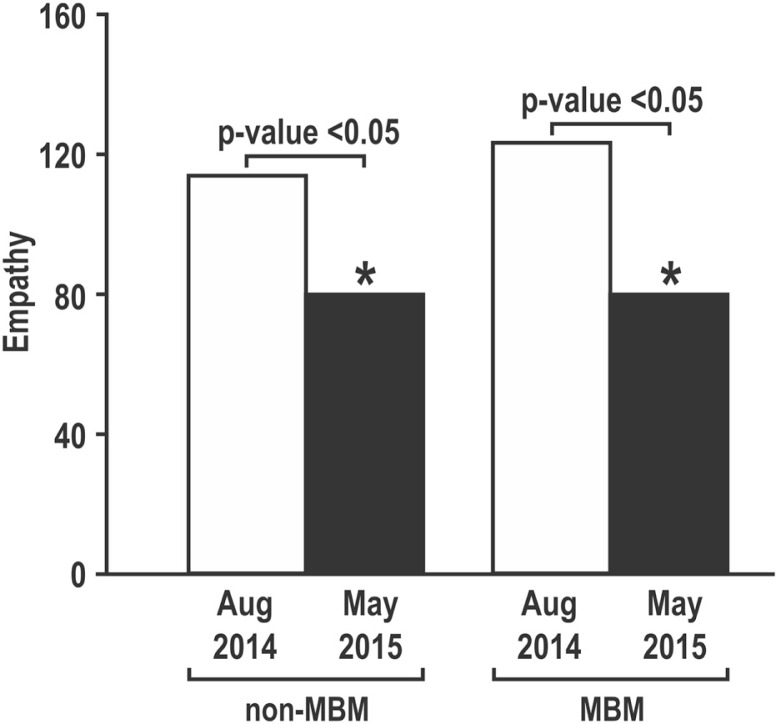
Scores on the Jefferson Scale of Physician Empathy-Student (JSPE-S) in first-year medical students.

As shown in Fig. 1, there was a statistically significant increase in PSS scores between the two time points in both groups. There were no statistically significant differences in either baseline or post-test stress scores between the two groups (*p*>0.05) (Fig. 1). Data regarding empathy are shown in Fig. 2. Both the control group and students who enrolled in the MBM course showed a statistically significant decrease in empathy (*p*<0.05). Here too, there was no significant difference in JSPE-S values between the two groups (*p*>0.05) either in baseline or post-test scores (Fig. 2).

**Fig. 3:**
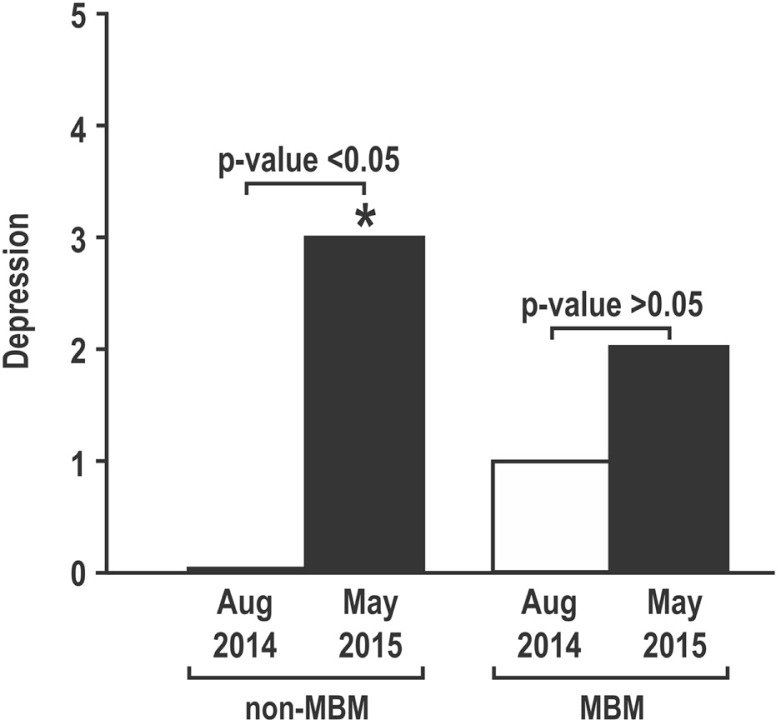
Scores on the Personal Health Questionnaire in first-year medical students.

With regard to the PHQ survey for depression (Fig. 3), there was a statistically significant increase in median values for depression in the control group (*p*<0.05), which was not seen in the students who took the MBM course.

## Discussion

Findings from this pilot study demonstrate that both control and MBM groups had statistically significant increases in stress levels during the course of the first year of medical school. It can be noted that the control, non-MBM group had PSS levels elevated to ‘high stress’ levels (>20), while the MBM group remained elevated but did not exceed that threshold considered ‘high stress’. However, the differences between the two post-tests were not statistically significant. Nevertheless, perceived stress levels were elevated among medical students regardless of participation in MBM.

The results reinforce the notion of an inverse relationship between increased stress and decreased empathy during medical training. We found that while stress increased significantly between pre- and post-test analysis for both MBM and non-MBM groups, empathy decreased significantly in the same time period for both groups (7).

Participation in the MBM course did not appear to alter the course of elevation in perceived stress and reduction in empathy. However, a difference was noted in the median depression scores. In the control group (non-MBM), median depression scores rose significantly, but did not change in the MBM group, suggesting a potential protective effect of an MBM course. It should be noted, however, that while the change was statistically significant, the PHQ scores in both groups were not in range of clinical depression.

The purpose of our study was to determine if a classroom intervention in the form of an MBM course might have an impact on stress, depression, and empathy. While we hypothesized that participation in the course might enable students to manage their stress better and boost their empathy, the findings of the small study failed to demonstrate those outcomes, perhaps related to the small sample size.

A major limitation of the study was the low rate of subject response. As a result of this, we were only able to analyze data on 20 subjects. As such, our study's power was 0.255 (after accounting for multiple comparisons). A further study with a higher sample size is hence needed to establish a statistical effect. Other limitations include non-uniformity of MBM courses offered throughout the country, making the results of the MBM course conducted at Georgetown University difficult to generalize to courses offered throughout the country. Perhaps MBM courses at Georgetown University are particularly adept to protective effects of depression and less impactful on stress and depression. Clearly, this pilot needs to be repeated with a larger sample over multiple years. Indeed, this study was conducted over the course of 1 year – the first year of medical school. It is important to follow students over the course of the 4-year curriculum and ideally compare students and programs at different institutions.

## Conclusion

Our study examined the impact of participation in an MBM course at Georgetown University School of Medicine on empathy, stress, and depression. The findings confirm that perceived stress increases and empathy declines during the first year of medical school. This inverse relationship was not altered by the MBM course participation. We did find that depression scores went up significantly in the control group but not in students who participated in the MBM course. While this pilot study was limited in scope, it does provide important information and will serve to provide baseline data for future studies examining the effects of MBM interventions in medical students.

## References

[1] Shapiro SL, Schwartz GE, Bonner G (1998). Effects of mindfulness-based stress reduction on medical and premedical students. J Behav Med.

[2] Mangione S, Kane GC, Caruso JW, Gonella JS, Nasca TJ, Hojat M (2002). Assessment of empathy in different years of internal medicine training. Med Teach.

[3] Suchman AL, Markakis K, Beckman H, Frankel R (1997). A model of empathetic communication in the medical interview. J Am Med Assoc.

[4] Helpern J (2003). What is empathy?. J Gen Intern Med.

[5] Firth-Cozens J, Greenhalgh J (1997). Doctors’ perceptions of the links between stress and lowered clinical care. J Soc Sci Med.

[6] Bellini LM, Baime M, Shea JA (2002). Variation of mood and empathy during internship. J Am Med Assoc.

[7] Thomas MR, Dybye LN, Huntington JL, Lawson KL, Novotny PJ, Sloan JA (2007). How do distress and well-being relate to medical student empathy? A multicenter study. J Gen Intern Med.

[8] MacLaughlin BW, Wang D, Noone AM, Liu N, Harazduk N, Lumpkin M (2011). Stress biomarkers in medical students participating in a mind body medicine skills program. Evid Based Complement Alternat Med.

[9] Lee J, Graham A (2001). Students’ perception of medical school stress and their evaluation of a wellness elective. Med Educ.

[10] Finkelstein C, Brownstein A, Scott C, Lan YL (2007). Anxiety and stress reduction in medical education: an Intervention. Med Educ.

[11] Karpowicz S, Harazduk N, Haramati A (2009). Using mind-body medicine for self-awareness and self-care in medical school. J Holist Health Care.

[12] Dutton MA, Arun P, Talley J, Haramati A, Harazduk N, Amri H (2013). Mind-body skills training for improving emotional well-being in medical students. Explore.

[13] Motz K, Graves K, Gross C, Saunders P, Amri H, Harazduk N (2012). OA05.03. Impact of mind body medicine skills course on medical students’ perceived stress, mindfulness, and elements of emotional intelligence. BMC Complement Altern Med.

[14] Harwani N, Motz K, Graves K, Amri H, Harazduk N, Haramati A (2013). Effect of a mind-body medicine skills course on perceived stress and empathy in medical students. Explore.

[15] Hojat M, Vergre MJ, Maxwell K, Brainard G, Herrine SK, Isenberg GA (2009). The devil is in the third year: a longitudinal study of erosion of empathy in medical school. Acad Med.

[16] Diseker RA, Michielutte R (1981). An anlysis of empathy in medical students before and following clinical experience. J Med Educ.

[17] Chang E, Eddins-Folensbee F, Coverdale J (2012). Survey of the prevalence of burnout, stress, depression, and the use of supports by medical students at one school. Acad Psychiatry.

[18] Mind Body Medicine Program Georgetown University, Department of Medical Education.

[19] Hojat M, Gonnella JS, Mangione S, Nasca TJ, Veloski FF, Erdmann FB (2002). Empathy in medical students as related to academic performance, clinical competence, and gender. Med Educ.

[20] Spitzer RL, Kroenke K, Williams J, and the Patient Health Questionnaire Primary Care Study Group (1999). Validation and utility of a self-report version of PRIME-MD: the PHQ primary care study. J Am Med Assoc.

[21] U.S. Preventive Services Task Force (2015). Final update summary: depression in adults: screening.

